# Evaluation of preoperative risk factors and postoperative indicators for anastomotic leak of minimally invasive McKeown esophagectomy: a single-center retrospective analysis

**DOI:** 10.1186/s13019-019-0864-4

**Published:** 2019-02-28

**Authors:** Chuan Gao, Gang Xu, Changyong Wang, Dong Wang

**Affiliations:** Department of Thoracic Surgery, Jinling Hospital, No.34 Yanggongjing, Qinhuai District, Nanjing, 210002 China

**Keywords:** Minimally invasive McKeown esophagectomy, Anastomotic leak, Diagnosis, Drainage amylase concentration

## Abstract

**Background:**

Minimally invasive McKeown esophagectomy is an important surgical approach for esophageal cancer. Anastomotic leak is one of its common and serious complications. We assumed that the preoperative risk factors and postoperative indicators would predict or detect anastomotic leak.

**Methods:**

Between December 2016 and July 2017, patients underwent minimally invasive McKeown esophagectomy were identified and their preoperative variables and postoperative test indicators were recorded. Fisher’s exact test, 2-tailed unpaired t test, nonparametric test and logistic regression were used to compare these datum between patients with or without anastomotic leak (AL). Receiver Operator Characteristic (ROC) curve was used to identify the best cut-off value of drainage amylase concentration for distinguishing anastomotic leak.

**Results:**

In all the 96 patients included, 12 patients were diagnosed as anastomotic leak by the esophagram. No differences in preoperative variables were observed between patients with and without AL. Patients in AL group appeared to have a lower prealbumin concentration in AL group on POD (postoperative day) 4(*P* = 0.05), POD 5(*P* = 0.04), POD 6 (*P* = 0.06). Prealbumin concentration cutoff value of 128 g/L on postoperative day 5 is 100.00% sensitive and 50.00% specific for predicting esophageal leaks. Drain amylases levels were higher in patients with anastomotic leak than those without anastomotic leak on POD 3(*P* = 0.03), POD 4(*P* = 0.01), POD 5(*P* < 0.001), POD 6(*P* < 0.001). The drain amylase cutoff value of 85 IU/L on postoperative day 4 was 75.00% sensitive and 84.00% specific for detecting esophageal leaks; the cutoff value of 65 IU/L on postoperative day 5 was 91.67% sensitive and 80.77% specific. The cutoff of 55/L on POD 6 is 100% sensitive and 86.96% specific.

**Conclusion:**

Drainage amylase concentration on postoperative days may help to discover anastomotic leak in early stage after minimally invasive McKeown esophagectomy. Prealbumin concentration below 128 g/L on POD 5 might be potential risk factor for anastomotic leak.

## Background

Anastomotic leak is a common and serious complication among esophagectomy patients [1, 2]. With the development of both surgical technique and perioperative management, the incidence of AL has decreased over these years, especially in minimally invasive esophagectomy, occurring in 2–2.96% of patients undergoing Ivor-Lewis esophagectomy while 6.6–13.64% receiving McKeown esophagectomy [3, 4]. The mortality rate of AL decreases approximately to 1.7% at experienced centers [5]. However, anastomotic leak is still a tough problem for thoracic surgery as it results in prolonged hospitalization psychological distress and even an increase in cancer recurrence [6]. Besides, as gastric conduit is widely applied, most esophageal anastomotic leak, small and latent with varied clinical symptoms in early stages, could be likely to result in abscess cavity or even systematic infection when be found out [7]. Thus, even the anastomosis is close to the cervical incision in McKeown esophagectomy, the signs of leak are not always obvious enough for early recognition and management, resulting in serious infection in neck, mediastinum and even chest [8, 9]. We have collected preoperative variables to identify which are risk factors for anastomotic leak. Meanwhile, the postoperative indicators were recorded and analyzed to assess whether they could detect or predict anastomotic leak.

## Methods

### Study population

Between December 2016 and July 2017, patients underwent minimally invasive McKeown esophagectomy for esophageal cancer at Department of thoracic surgery in Jinling Hospital were identified. Approved by the Ethics Committee of the institution, we obtained written informed consents from all patients**.**

### Data collection

Patient demographic information, comorbidity (hypertension, diabetes, and COPD), drink history, tumor stage, history of received neoadjuvant chemoradiation were collected preoperatively and types of anastomosis, types of enteral nutrition (nasojejunal tube or jejunostomy tube), anastomotic methods were recorded postoperatively. We collected the pleural fluid from the chest tube and measured its amylase concentration from 1 to 6 postoperative days (POD) in all the 96 patients. Besides, temperature, heart rate, prealbumin concentration, white and red blood cell count were also measure. All the information was recorded in time from each patient.

### Clinical definition and procedure

Anastomotic leak was defined as a gastroesophageal defect involving esophagus, anastomosis and conduit. All patients had been screened by upper gastroenterogram before they took in food. The treatments were same whether the leak was from anastomosis or gastric conduit according to our experience. Therefore, gastric conduit necrosis was also classified into anastomotic leak in our study. In other studies, the two complications were also generally called gastric conduit failure [10, 11]. Jejunostomy tube was suggested among patients who were less tolerable with odynophagia or minded about self-image. Nasojejunal feeding was preferred for those who may need supplement enteral nutrition before operation or with severe intestinal adhesion met in operation. Chest tube was placed in the esophageal groove up to the right thoracic inlet. Amylase of drainage fluid was monitored from POD 1 to POD 6 unless the anastomotic leak was affirmed beforehand. Esophagram was normally conducted with gastrografin on POD 7. Only the drainage fluid located in the chest tube was sampled so that amylase concentration was a real-time reflection of pleural fluid.

### Statistical analysis

Datum were tested for normality and presented as mean and standard deviation or median and interquartile range where appropriate. Fisher’s exact test, 2-tailed unpaired t test, nonparametric test and logistic regression were used to compare the patient characteristics, preoperative variables and postoperative indicators between patients with AL and those without. Receiver Operator Characteristic (ROC) curve was used to identify the best cut-off value of drainage amylase concentration for distinguishing anastomotic leak. *P* < 0.05 was considered statistically significant. Statistical analysis was performed using SPSS 19.0 and SigmaPlot 13.0.

## Results

There are totally 96 patients who meet the inclusion criteria between December 2016 and July 2017. 69 patients are male and 27 are female, accounting for 71.88 and 28.12% respectively. The mean age is 62 (range: 45–84). 71 patients received jejunostomy while 25 patients accepted nasojejunal tubes.12 patients were as diagnosed as anastomotic leak by esophagram (12.5%). In order to distinguish the risk factors among the patient characteristics for anastomotic leak, the patients were divided into two groups according to whether they suffered from anastomotic leakage. As showed in Table 1, there is no difference in sex, age and drinking history between two groups. No significant difference has been found between comorbidity groups with hypertension, diabetes and COPD and tumor staging groups. Among the factors related to therapeutic intervention including neoadjuvant therapy, feeding types and anastomotic methods, no statistical differences are observed either.

**Table 1:** Patient characteristics

Variable	Anastomotic leakage (*n* = 12)	None anastomotic leakage (*n* = 84)	Univariate *P*	Multivariate *P*
Sex			0.35	0.24
Male	10	59		
Female	2	25		
Age	63.42 ± 9.13	62.02 ± 8.48	0.60	0.71
Comorbidity			0.43	0.48
None	6	47		
Hypertension	6	26		
Diabetes	0	7		
COPD	0	4		
Drinking history			0.43	0.66
Yes	6	32		
No	6	52		
Staging			0.91^*^	0.87
IA/IB/IIA/IIB/IIIA/IIIB/IIIC	1/2/2/3/3/1/0	4/7/16/25/16/13/3		
Neoadjuvant therapy			0.88	0.71
Yes	5	33		
No	7	51		
Feeding type			0.19	0.27
Jejunostomy	7	64		
Nasojejunal tubes	5	20		
Anastomotic methods			0.65	0.82
Handsewn	2	7		
Linear stapled	5	38		
Circular stapled	5	39		

*Tested by Mann-Whitney U test

As described in Table 2, there is no significant difference in temperature, heart rate, chest drainage, WBC count, RBC count, urine volume between AL and None AL groups on postoperative days. Patients in AL group appear to have a lower prealbumin concentration on POD 4(*P* = 0.05), POD 5(*P* = 0.04), POD 6 (*P* = 0.06). There is no significant difference in Drain amylases in both groups on POD 1 and POD 2, but the levels are higher in patients with anastomotic leak than those without anastomotic leak on POD 3(*P* = 0.03), POD 4(*P* = 0.01), POD 5(*P* < 0.001), POD 6(*P* < 0.001).

**Table 2:** Postoperative indicators for anastomotic leak

Postoperative indicators	POD 1	POD 2	POD 3	POD 4	POD 5	POD 6
Temperature(*t*/*P* value)	0.169/0.867	−0.411/0.684	2.31/0.03	0.70/0.49	0.31/0.76	−0.129/0.9
Heart rate(*t*/*P* value)	0.14/0.89	−1.24/0.22	−0.60/0.55	− 0.11/0.91	− 0.02/0.99	− 0.29/0.77
Urine volume(*t*/*P* value)	0.82/0.42	− 0.034/0.97	− 0.941/0.35	−1.93/0.06	− 0.14/0.89	2.09/0.05
Prealbumin(*t*/*P* value)	−0.775/0.45	− 0.732/0.51	− 0.90/0.38	−2.47/0.05	−2.27/0.04	−2.08/0.06
Chest drainage volume(*t*/*P* value)	−1.329/0.2	− 0.38/0.71	0.18/0.86	0.23/0.82	−0.58/0.57	− 0.85/0.4
WBC count(*t*/*P* value)	0.31/0.76	−0.60/0.56	−0.38/0.71	0.54/0.6	0.48/0.64	−0.14/0.89
RBC count(*t*/*P* value)	0.32/0.75	0.52/0.62	−0.85/0.4	0.81/0.44	−0.37/0.71	−0.72/0.48
Drainage amylase (Z/*P* value)	−0.773/0.46	−0.70/0.49	−2.13/0.03	− 2.46/0.01	−3.72/< 0.001	−3.34/< 0.001

**Fig. 1:**
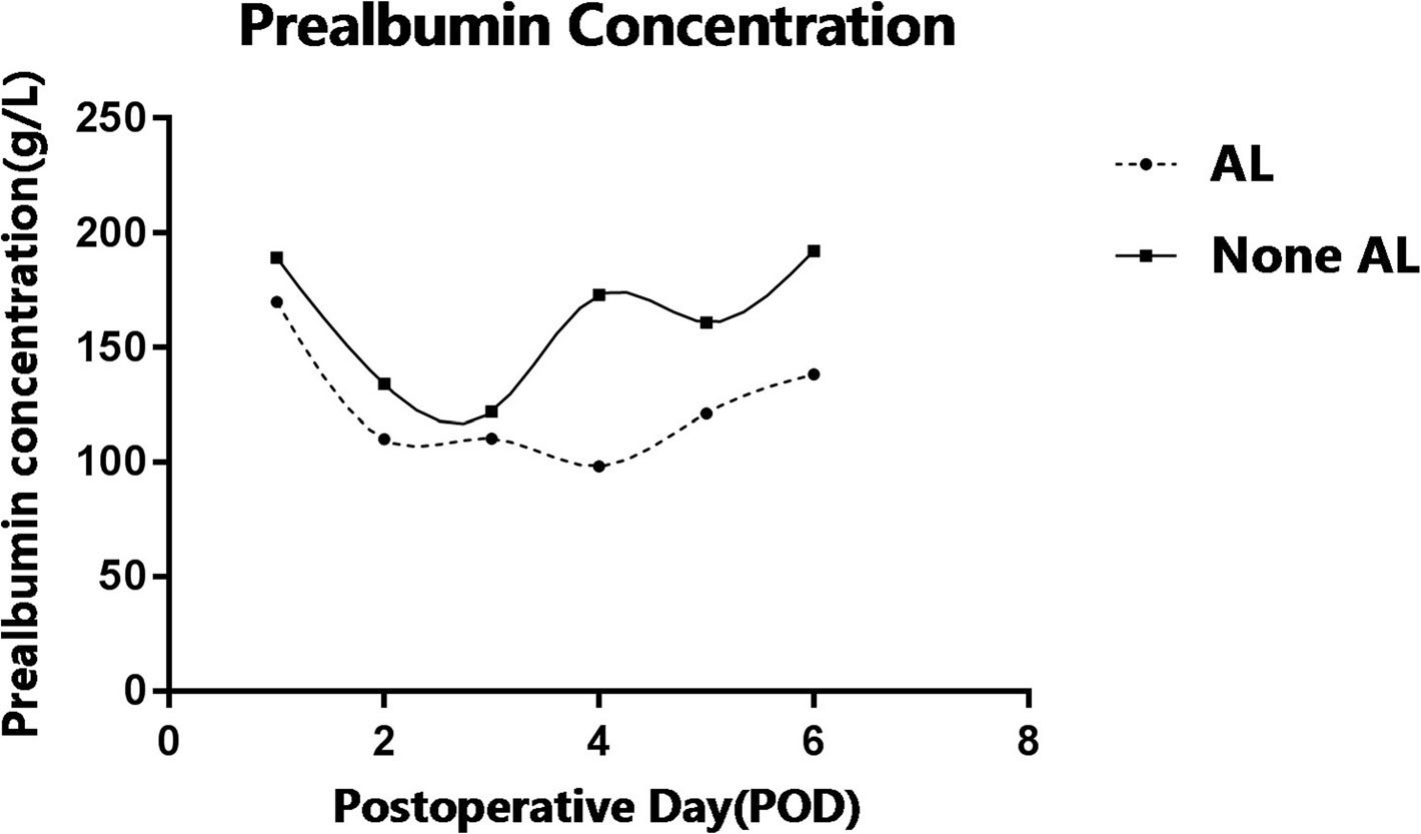
Serum prealbumin concentration trend on postoperative day in AL and None AL group. The concentration of prealbumin between AL group and None AL group appears to show significant difference on POD 4(t = − 2.47, P = 0.05), POD 5(t = − 2.27, P = 0.04), POD 6 (t = − 2.08, *P* = 0.05). The AL group has a lower concentration of prealbumin while the trends of prealbumin on postoperative day in both groups are similar. Serum prealbumin concentration cutoff value of 128 g/L on postoperative day 5 is 100.00% sensitive and 50.00% specific for detecting esophageal leaks

The prealbumin concentration in both AL groups and None AL group exhibit a significant decrease since POD 1. This decreasing trend starts rebounding from POD 3 in None AL group while the rise in AL group is slower and gentler. The difference between two groups begin to be obvious from POD 4 but diminishes on POD 6 (Fig. 1). According to the results in Table 2, serum prealbumin on POD 5 is statistically significant between None AL and AL group. The serum prealbumin concentrations are analyzed by ROC curve and the cutoff value of 128 g/L on postoperative day 5 is 100.00% sensitive and 50.00% specific for detecting esophageal leaks. The AUC area is 0.825(*P* = 0.046).

**Fig. 2:**
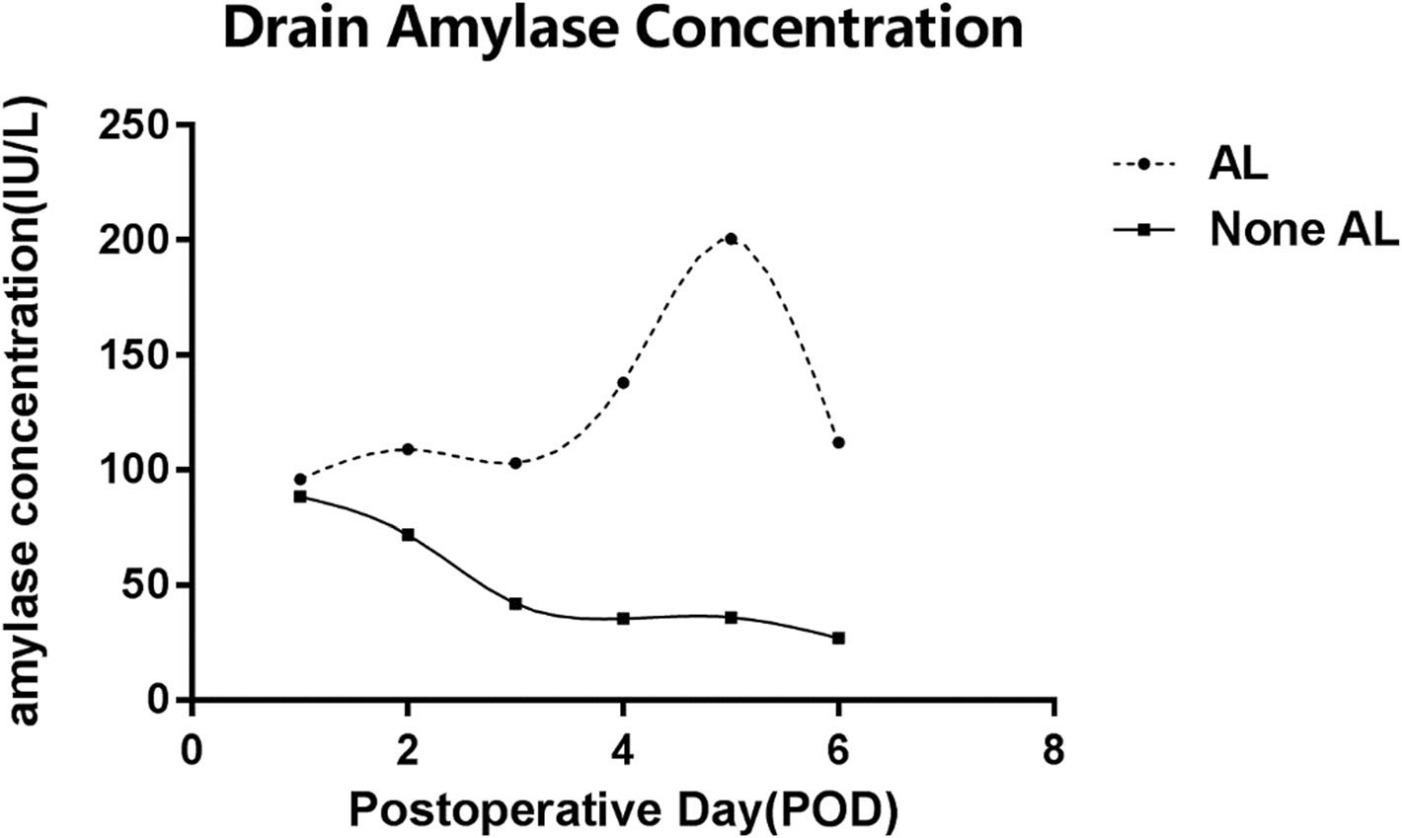
Drain amylase concentration trend on postoperative days in AL and None AL group. The concentration of drain amylase between AL and None AL group show significant difference on POD 3(Z = -2.13, P = 0.03), POD 4(Z = -2.46, P = 0.01), POD 5(Z = -3.72, P < 0.001) POD 6(Z = -3.34, *P* < 0.001)

Patients in both AL group and None AL group have similar drainage amylase concentration on POD 1. In the None AL group, the drainage amylase levels decline gradually in the following postoperative day. However, in the AL group, this trend starts with a slow rise in the first three postoperative days and shows an obvious growth on the following 2 days followed by a sharp decline on POD 6. The discrepancy between groups is significant from POD 3 to POD 6 consequently (Fig. 2).

**Fig. 3:**
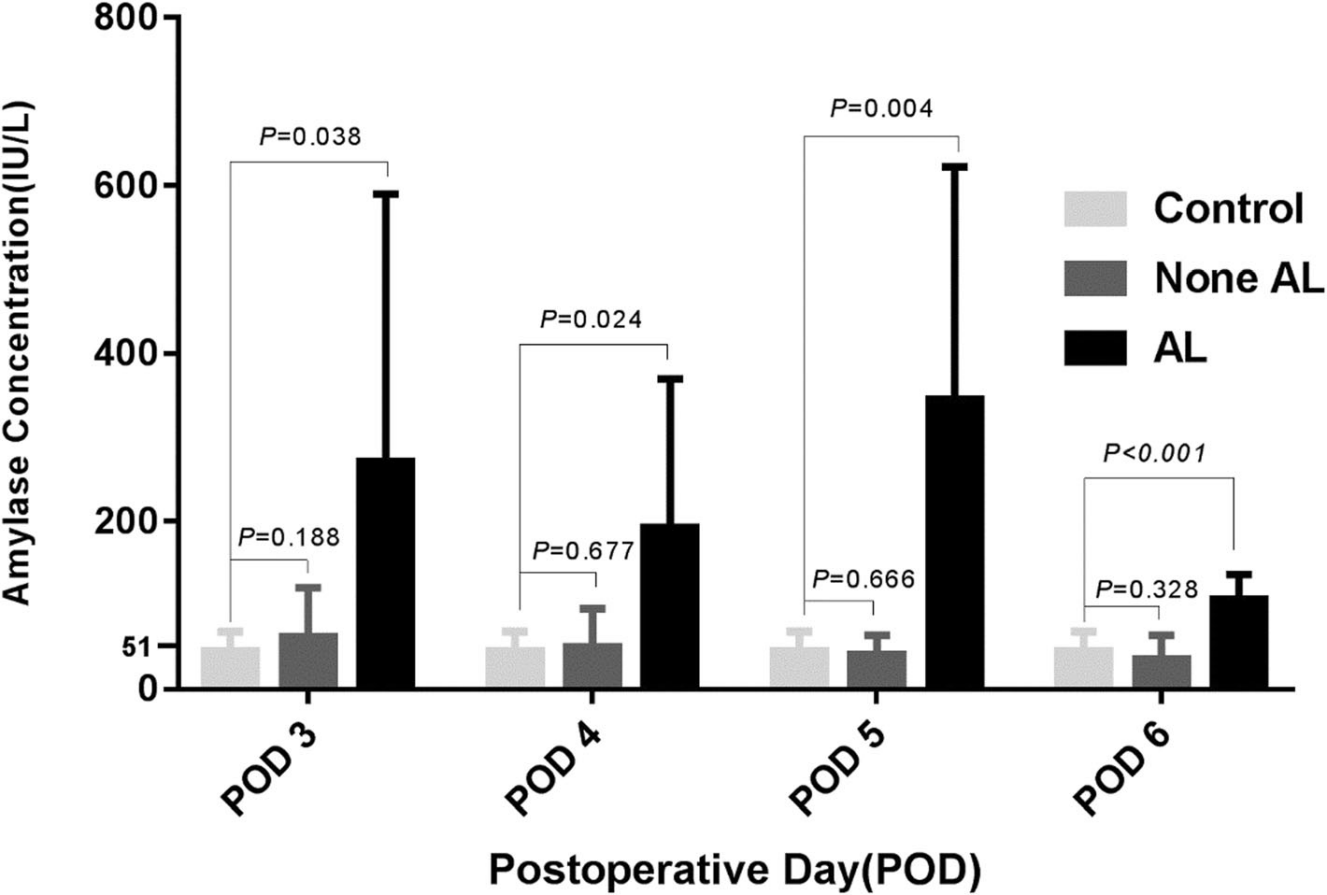
Contrast of the average values of drain amylase concentration on postoperative days there’s no statistical distinction in the amylase concentration between None AL group and control group. However, the difference between AL group and control group is significant

We also tested drain amylase concentration in the control group which is populated by patients receiving lobectomy or segmentectomy to determine whether drain amylase in AL group mainly comes from saliva (Fig. 3). As the chest tube is extubated 2 or 3 days after pulmonary surgery, we obtained the drain average amylase concentration on POD 1 and POD 2. By comparing the average values of drain amylase in control group to those in None AL and AL group respectively, we find that there’s no statistical distinction in the amylase concentration between None AL group and control group. The difference between AL group and control group, however, is significant.

**Fig. 4:**
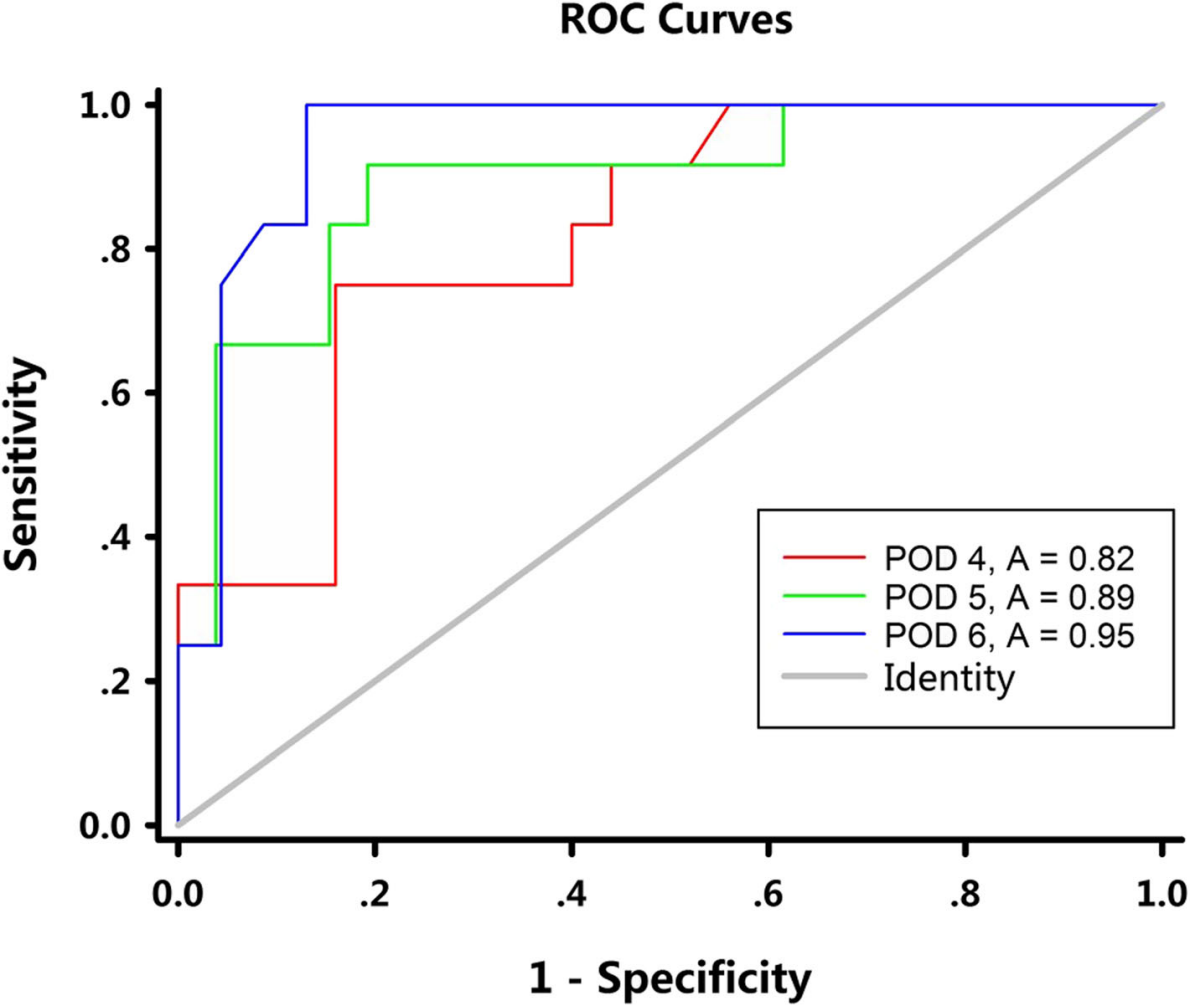
Receiver operating characteristic (ROC) for the association of drain amylase and anastomotic leak on POD 4, POD 5, POD 6. The cutoff of 85 IU/L on POD 4 is 75.00% sensitive and 84.00% specific (AUC area = 0.82, *P* = 0.002). The cutoff of 65 IU/L on POD 5 is 91.67% sensitive and 80.77% specific (AUC area = 0.89, *P* = 0.0001). The cutoff of 55/L on POD 6 is 100% sensitive and 86.96% specific (AUC area = 0.95, *P* < 0.0001)

The drainage amylase concentrations on all postoperative days are analyzed by ROC curve. The area under curve (AUC) are respectively 0.60(*P* = 0.4398), 0.59 (*P* = 0.4839), 0.64(*P* = 0.1670) for POD 1, POD 2, POD 3. Figure 4 represents the ROC curve for drainage amylase on POD 4, POD 5 and POD 6. The drain amylase cutoff value of 85 IU/L on postoperative day 4 is 75.00% sensitive and 84.00% specific for detecting esophageal leaks. On postoperative day 5, the cutoff value of 65 IU/L is 91.67% sensitive and 80.77% specific. On postoperative day 6, the cutoff value of 55 IU/L is 100% sensitive and 86.96% specific. The *P* value calculated by Delong’s method [12] for comparison of the AUC between POD 4 and POD 5, POD 4 and POD 6, POD 5 and POD 6 are 0.299, 0.061 and 0.361 respectively, indicating that these three cutoff values have similar sensitivity and specificity in predicting anastomotic leak.

## Discussion

Minimally invasive esophagectomy becomes popularized rapidly, which means it limits tissue trauma without compromising oncologic resection [5, 13, 14]. However, as gastric conduit is routinely used for replacement of esophagus, gastric conduit failure appears to be more common than previous open surgery [10]. Besides, patients receiving minimally invasive McKeown esophagectomy are more vulnerable to gastric conduit failure than patients who received minimally invasive Ivor-Lewis esophagectomy [15, 16]. Once the anastomosis or the tip of gastric conduit undergoes ischemia, it may finally progress to anastomotic leak over a period. As crevice increases in size, the symptom develops from mild to serious. On early detection, this complication could be treated timely to avoid serious consequences.

Amylase is a digestive enzyme found in low concentration in blood (< 140 IU/L) but high in saliva (70,000 IU/L) [17]. The drainage amylase level has been used to diagnose anastomotic leak in laryngectomy, pancreatectomy, gastrectomy and Roux-en-Y procedures in past decades [18–20]. Thus, it is a reasonable hypothesis that amylase could also be used as an indicator for esophageal anastomotic leak by testing the concentration of the drainage fluid following esophagectomy [21, 22]. But in previous studies, the placement of drainage tube was high-demanding: locating next to the anastomosis with a specialized tube. Detecting anastomotic leak by drainage amylase for minimally invasive McKeown esophagectomy has never been recorded before. In the past we routinely put a drainage strip in the neck incision and removed it in 48 h, which was convenient for detecting anastomotic leak. However, anastomotic leak often occurs after POD 3 according to our experience.

The drainage tubes in our study were placed in the chest instead of next to the anastomosis by neck incision. We suppose the amylase of drainage fluid is also sensitive to anastomotic leak. Since the thoracic cavity pressure is negative, once the anastomotic leak occurs, saliva could be sucked into chest. By the movements of the breath, the drainage fluid would be contaminated.

**Fig. 5:**
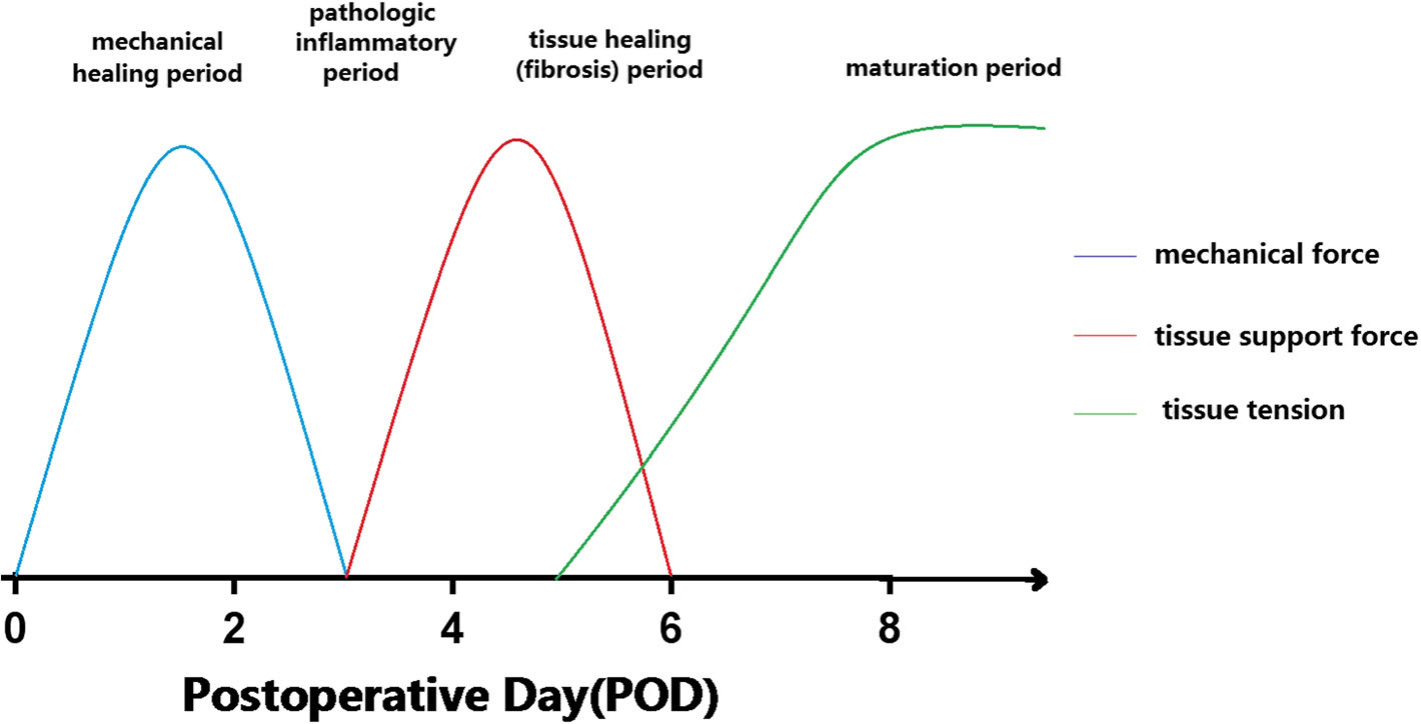
The course of digestive tract reconstructions. POD 3–5 is just in the pathologic inflammatory period when the joint is weak and most anastomotic leaks occur

Our study found that amylase cutoff of 55 IU/L on POD 6 was 100% sensitive and 86.96% specific in detecting anastomotic leak, and the corresponding AUC was the largest. However, there is no significant difference among the AUCs of POD 4, POD 5 and POD 6. According to principle of early diagnosis and early intervention, amylase cutoff value of 85 IU/L on POD 4 or 65 IU/L on POD 5 has higher diagnostic value. When these test results come along with symptoms such as fever, chest or incision pain and dyspnea, anastomotic leak should be taken into consideration. The reason for the difference of amylase between AL group and None AL group begins to show itself from POD 4 may be related with the course of digestive tract reconstruction. Doctor HU Xiang has divided this course into four stages: mechanical healing period(1-3d), pathologic inflammatory period(3-5d), tissue healing (fibrosis) period(5-7d) and maturation period(7d-later) [23]. In mechanical healing period, the joint of anastomosis depends on the stapler nails or sutures. When it comes to the inflammatory period, the joint begins to be strengthened by the tissue support force. Then in the healing (fibrosis) period granulation tissue starts proliferating and inflammatory cells are subsiding, causing mucous epithelium cells to grow and cover the anastomosis. After 7 days, the digestive tract reconstruction completes. POD 3–5 are just in the pathologic inflammatory period when the joint is weak and most anastomotic leaks occur (Fig. 5). Besides, gastric conduit necrosis is also an important factor causing anastomotic leak [5]. Doctor Darmarajah Veeramootoo [10] had researched on the relationship between gastric conduit failure and the postoperative C-reactive protein level. He also found that elevated CRP levels in the absence of any other clinical cause beyond POD 3 raises suspicion of incipient gastric conduit failure. Above all, POD 3–5 is a crucial period for doctors to recognize anastomotic leak and conduct appropriate management immediately before the impact of contamination develops. However, it’s supposed to be unsafe to received esophagram in this period, whereas drainage amylase is a good choice.

To confirm that the rise of drain amylase concentration is attributed to saliva from esophagus instead of pleural effusion, we collect the drain fluid from patients receiving pulmonary surgery on POD 1 and POD 2. We found that the drain amylase concentration in AL group is not only significantly higher than that in None AL group but also higher than the pleural fluid from patients of pulmonary surgery, while the levels of amylase concentration in both pulmonary surgery group and None AL group are similar. This result verifies our supposition that the distinct rise of drain amylase concentration in AL group is due to saliva, a fluid full of amylase [24], which mixes into pleural fluid.

Besides, the drop of drain amylase concentration on POD 6 in AL group draws our attention. We think that the significant decline of amylase level may be attributed to the clinical intervention. Many patients may have clinical symptoms such as fever, incision pain or elevation in white blood cell on POD 5 or POD 6 due to anastomotic leak. Therefore, treatments like incision flush and drainage may be conducted and the level of drainage amylase concentration consequently declines. These factors should be considered when interpreting our data. So, the relevance between the incidence rate of anastomotic leak and drainage amylase concentration may decline on POD 6.

Prealbumin is a protein synthesized in the liver, metabolized and excreted by the kidneys. As it has a short half-life, its serum level change rapidly in response to nutritional status. Once the patients got hypoproteinemia, the gastric conduit and bowel mucosa may appear edema consequently, which would worsen the absorption of nutrition and finally lead to anastomotic leak. Our results revealed that patients in AL tend to show lower prealbumin since POD 4 to POD 6. It’s understandable that surgery may significantly influence nutrient metabolism. In Ying-Jian Wang et al’s study [25], they recorded the preoperative and postoperative levels of albumin and prealbumin of patients who underwent MIE(minimally invasive McKeown esophagectomy). They found that the decrease of prealbumin after MIE was associated with the incidence of cervical AL. Interestingly, albumin was not observed to have such relationship with AL. Our study found that patients may have high risk of AL if their serum prealbumin concentration was below 128 g/L on postoperative day 5. However, low prealbumin concentration can’t reflect AL promptly, it may not be sensitive enough as an indicator of AL.

In our study, we also screened the risk factors of esophageal anastomotic leak among the patients’ characteristics, tumor features and therapeutic methods. Although previous studies have reported several hazards for AL [3, 26–28], we found no statistically significant risk factors in our study. As a retrospective study in single center with small quantity of patients, our study may have potential bias. It may reduce the reliability of cutoff value. Therefore, further prospective studies are needed to be done.

## Conclusion

In summary, our study reveals that drainage amylase concentration on postoperative days may help to discover anastomotic leak in early stages after minimally invasive McKeown esophagectomy. Patients with amylase level above 85 IU/L on POD 4 or 65 IU on POD 5 should be considered suffering anastomotic leak especially when they have symptoms such as fever, incision or thoracic pain. Besides, prealbumin concentration below 128 g/L on POD 5 might be potential risk factor for anastomotic leak.

## Abbreviations

AL: Anastomotic leak
AUC: Area under curve
COPD: Chronic obstructive pulmonary disease
MIE: Minimally invasive McKeown esophagectomy
None AL: None Anastomotic leak
POD: Postoperative day
ROC: Receiver Operator Characteristic
